# FZD5 prevents epithelial-mesenchymal transition in gastric cancer

**DOI:** 10.1186/s12964-021-00708-z

**Published:** 2021-02-22

**Authors:** Dan Dong, Lei Na, Kailing Zhou, Zhuo Wang, Yu Sun, Qianqian Zheng, Jian Gao, Chenghai Zhao, Wei Wang

**Affiliations:** 1 Department of Pathophysiology, College of Basic Medical Science, China Medical University, Shenyang, People’s Republic of China; 2 Department of Urology, Shengjing Hospital of China Medical University, Shenyang, People’s Republic of China; 3 Center of Laboratory Technology and Experimental Medicine, China Medical University, Shenyang, People’s Republic of China

**Keywords:** FZD5, Epithelial-mesenchymal transition, ELF3, Gastric cancer

## Abstract

**Background:**

Frizzled (FZD) proteins function as receptors for WNT ligands. Members in FZD family including FZD2, FZD4, FZD7, FZD8 and FZD10 have been demonstrated to mediate cancer cell epithelial-mesenchymal transition (EMT).

**Methods:**

CCLE and TCGA databases were interrogated to reveal the association of FZD5 with EMT. EMT was analyzed by investigating the alterations in CDH1 (E-cadherin), VIM (Vimentin) and ZEB1 expression, cell migration and cell morphology. Transcriptional modulation was determined by ChIP in combination with Real-time PCR. Survival was analyzed by Kaplan–Meier method.

**Results:**

In contrast to other FZDs, FZD5 was identified to prevent EMT in gastric cancer. FZD5 maintains epithelial-like phenotype and is negatively modulated by transcription factors SNAI2 and TEAD1. Epithelial-specific factor ELF3 is a downstream effecter, and protein kinase C (PKC) links FZD5 to ELF3. ELF3 represses ZEB1 expression, further guarding against EMT. Moreover, FZD5 signaling requires its co-receptor LRP5 and WNT7B is a putative ligand for FZD5. FZD5 and ELF3 are associated with longer survival, whereas SNAI2 and TEAD1 are associated with shorter survival.

**Conclusions:**

Taken together, FZD5-ELF3 signaling blocks EMT, and plays a potential tumor-suppressing role in gastric cancer.

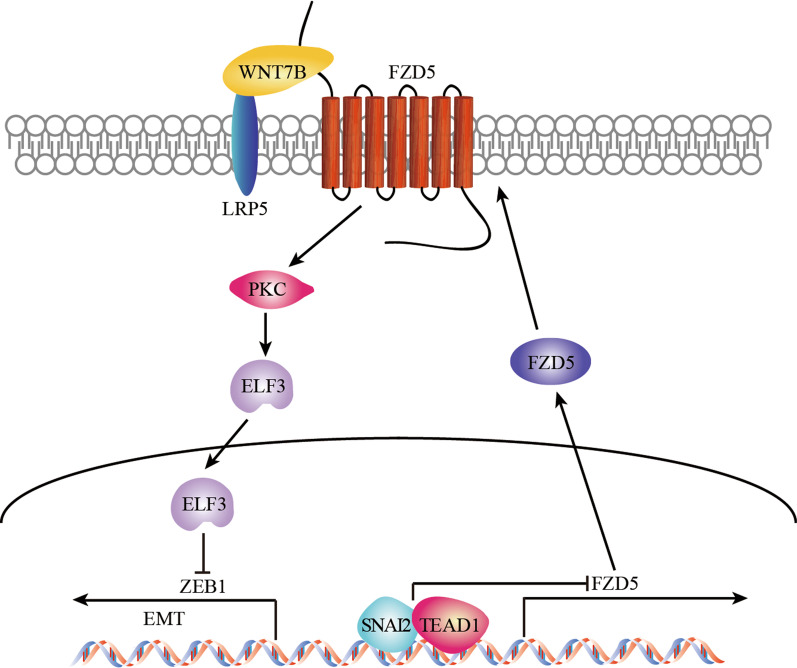

**Video Abstract**

## Background

Frizzled (FZD) proteins are G protein-coupled receptors (GPCRs). The extracellular N-terminus contains a cysteine-rich domain (CRD) through which FZDs bind WNT ligands. The intracellular C-terminus binds the PDZ domain of Dishevelled (Dvl) and interacts with G proteins. Up to now, 10 FZDs have been identified in human. These receptors mediate β-catenin pathway and various β-catenin-independent pathways depending on cellular context. WNT/β-catenin pathway is oncogenic and involved in almost every aspect of tumor development. In addition, β-catenin-independent WNT/PCP and WNT/Ca^2+^-PKC pathways are also implicated in tumor progression [1–4]. In recent years, some novel pathways such as WNT/Stat3 and WNT/Yap-Taz have been successively identified [5–8], indicating that FZD signalings are far more complicated and still incompletely comprehended.

Epithelial-mesenchymal transition (EMT) in tumor cells endows them with enhanced motility thereby increasing their metastatic potentiality. This process is characterized by reduced expression of epithelial-related factors and increased expression of mesenchymal-related factors. Mechanistically, EMT is driven by some transcription factors such as SNAI1/2 and ZEB1/2. Studies have shown that FZD signalings induce tumor cell EMT and metastasis. FZD4, FZD7 and FZD10 promote EMT through β-catenin pathway in prostate, liver and breast cancer [9–11]. FZD2 mediates WNT5-induced EMT dependent on Stat3 signaling in liver, lung, breast and colon cancer [5], and FZD8 mediates WNT11-induced EMT by interacting with TGF-β signaling in prostate cancer [12].

In gastric cancer, FZD4 and FZD7 similarly promote EMT and metastasis by activating β-catenin pathway [13, 14]. Genetic deletion of FZD7 was shown to inhibit the growth of gastric adenomas in vivo [15]. FZD2 and FZD8, through β-catenin pathway but not β-catenin-independent pathways, induce gastric cancer cell proliferation, migration, invasion and metastasis [16, 17]. Unexpectedly our study uncovered that FZD5 prevents EMT and is associated with longer survival in gastric cancer, suggesting FZD5 as a putative suppressor in this type of cancer.

## Methods

### In silico analysis

Cancer Cell Line Encyclopedia (CCLE) database was interrogated for gene expression in 37 human gastric cancer cell lines. Correlation between two genes was analyzed by Pearson statistics. The Cancer Genome Atlas (TCGA) database was interrogated for GO pathway enrichment analysis in 375 gastric cancer tissues and 32 normal gastric tissues. GSE62254 database was interrogated in Kaplan Meier plotter website (http://kmplot.com/analysis/) to assess the survival [18, 19].

### Cell culture and transfection

Human gastric cancer cell line MKN45 and HGC27 were cultured in RPMI 1640 medium supplemented with 10% fetal bovine serum in a humidified incubator at 37 °C with 5% CO_2_. 5 × 10^4^ MKN45 cells were transfected with shRNA lentiviruses to stably knockdown FZD5 expression; 5 × 10^4^ HGC27 cells were transfected with overexpression lentiviruses to stably overexpress FZD5, and the transfected cells were further selected using 2 μg/ml puromycin. For transient transfection, 2.5 × 10^5^ cells were transfected with shRNA or overexpression plasmids using Lipofectamine 3000 in Opti-MEM medium according to the product manual. Target sequences for shRNA were listed in Table 1.

**Table 1:** Target sequences for shRNA

Genes	Target sequences (5′ to 3′)
FZD5 shRNA-1	GGCCACCTTCCTCATCGACAT
FZD5 shRNA-2	CGGCATCTTCACGCTGCTCTA
SNAI2 shRNA-1	CCCATTCTGATGTAAAGAAAT
SNAI2 shRNA-2	CCTCACTGCAACAGAGCATTT
TEAD1 shRNA-1	CCGATTTGTATACCGAATAAA
TEAD1 shRNA-2	CCATTCTTACAGTGACCCATT
ELF3 shRNA-1	CAACTACTTCAGTGCGATGTA
ELF3 shRNA-2	GCCATGAGGTACTACTACAAA
LRP5 shRNA-1	GAGGAGCTACTTCCATCTCTT
LRP5 shRNA-2	CTCCCACATCTGTATTGCCAA
WNT7B shRNA-1	GCGCCTCATGAACCTGCATAA
WNT7B shRNA-2	CGTGCGTTACGGCATCGACTT

### Western blot

SDS-PAGE gel was used to isolate proteins in whole cell lysate. Proteins were transferred to PVDF membrane which was blocked with 5% bovine serum albumin, and incubated with the indicated primary antibodies. The membranes were than incubated with peroxidase-conjugated goat anti-rabbit or peroxidase-conjugated goat anti-mouse (ZSGB Bio) secondary antibody (1:5000). Protein content was detected with SuperSignal™ West Pico PLUS Chemiluminescent Substrate (Thermo Scientific). Primary antibodies for FZD5 (1:1000), SNAI2 (1:1000), LRP5 (1:1000) were purchased from Cell Signaling Technology. Primary antibody for ELF3 (1:1000) was provided by Thermo Scientific. Primary antibodies for TEAD1 (1:2000) and WNT7B (1:1000) were obtained from Abcam.

### Real-time PCR

RNAiso Plus (Takara) was used to extract total RNAs, which were then reversely transcribed into cDNA using PrimeScript™ RT reagent Kit with gDNA Eraser (TaKaRa)according to the instructions. Real-time PCR was performed using TB Green™ *Premix Ex Taq*™ II (TaKaRa). Primers for genes and transcription factor binding sequences were listed in Table 2. GAPDH was used as endogenous control. Expression difference was analyzed using 2^−ΔΔCT^ method.

**Table 2:** Primers for Real-time PCR

Genes or binding sites	Primer (5′ to 3′)
CDH1-forward	GCCATCGCTTACACCATCCTCAG
CDH1-reverse	CTCTCTCGGTCCAGCCCAGTG
ELF3-forward	ATGGTTTTCGTGACTGCAAGAA
ELF3-reverse	CAGTACTCTTTGCTCAGCTTTC
FZD5-forward	TCCTCTGCATGGATTACAACC
FZD5-reverse	GACACTTGCACACGAACG
GAPDH-forward	CAGGAGGCATTGCTGATGAT
GAPDH-reverse	GAAGGCTGGGGCTCATTT
LRP5-forward	CCCTACATCATTCGAGGAATGG
LRP5-reverse	CCGAGTTCAAATCCAGGTAGTA
VIM-forward	GGTGGACCAGCTAACCAACG
VIM-reverse	TTGCAGGGTGTTTTCGGCTT
ZEB1-forward	CAGGCAAAGTAAATATCCCTGC
ZEB1-reverse	GGTAAAACTGGGGAGTTAGTCA
SNAI2 BS1-forward	ATTTGGGCGGCTACTATGT
SNAI2 BS1-reverse	CCTTACAACAATTCTGAGGC
SNAI2 BS2-forward	ACCTGCCTCAGAATTGTTG
SNAI2 BS2-reverse	GAACTCCTGACACCGTGAT
TEAD1 BS1-forward	GTCCAGTCCCTATCCCTT
TEAD1 BS1-reverse	AAATGCCTCTTTTCTATGTG
TEAD1 BS2-forward	GGCAGGATAATCGCTTGA
TEAD1 BS2-reverse	GGATACTTACTGAGCCCTTTAC

### Immunofluorescence

Cells were fixed with 4% paraformaldehyde, and incubated with PBS containing 0.1% Triton X-100. Slices were then incubated with primary antibodies for CDH1 (1:200, Cell Signaling Technology) and VIM (1:100, Cell Signaling Technology), and subsequently incubated with Alexa 488 Donkey anti-Rabbit IgG secondary antibody (1:1000, Invitrogen). The fluorescence was visualized by a confocal microscope.

### Phalloidin staining

Cells on the slides were fixed with 4% formaldehyde at room temperature for 10 min. Then the slides were washed with PBS 3 times, and incubated with 0.5% Triton X-100. TRITC-conjugated Phalloidin solution (YEASEN, Shanghai, China) was added on the slides incubated at room temperature for 30 min. DAPI was used to stain the nuclei. Finally, cell morphology was visualized by a confocal microscope.

### Cell migration

Transwell chambers were used to determine cell migration ability. 2 × 10^4^ cells were added into the upper chamber. Culture medium with 10% FBS was added to the lower chamber. After 24 h, non-migrated cells were removed with a swab. The chambers were fixed with 4% paraformaldehyde for 15 min at room temperature, and incubated with 0.1% crystal violet for 30 min at 37℃. Cells were determined in 5 randomly selected microscope fields.

### Chromatin immunoprecipitation (ChIP)

A ChIP assay kit (Beyotime, China) was used according to the manufacturer’s instructions. Briefly, Cells in 10 cm plates were fixed with 1% formaldehyde for 10 min at 37 °C. To shear the chromatin, cells were treated with 1 mM PMSF in SDS Lysis Buffer for 10 min at 4 °C, followed by cell sonication for 15 min at 4 °C. After a portion of the cross-linked chromatin was removed as input for the subsequent test, the remaining cell lysis was incubated with 1 μg primary antibody at 4 °C overnight. Then protein A + G agarose was added to precipitate the target protein recognized by the primary antibody for 1 h at 4 °C. IgG antibody was used as a negative control. The beads were then washed off and DNA was collected for subsequent Real-time PCR assays. The enrichment was indicated as % of input.

### Co-immunoprecipitation (CoIP)

500 µg protein was incubated with 2 µg primary antibody at 4 °C for 4 h. 20 µl Protein A/G PLUS Agarose beads (Santa Cruz Biotechnology) were added at 4 °C overnight. Then the agarose beads were washed to clear non-specifically bound proteins. The target protein was detected by Western blot.

### Human specimens

55 gastric cancer tissues were obtained from Shengjing Hospital China Medical University with the informed consent of the patients. Institutional Research Ethics Committee of China Medical University approved the use of these tissues for research purposes.

### Immunohistochemistry

Paraffin-embedded sections were deparaffinized and rehydrated. Sections were in turn added endogenous peroxidase blockers, normal non-immune animal serum, primary antibody, biotin–conjugated secondary antibody, streptomycin antibiotic-peroxidase solution, freshly prepared DAB. Then sections were counterstained with Mayer’s hematoxylin, dehydrated, cleared and mounted. Primary antibodies for FZD5 (1:100) and TEAD1 (1:200) was purchased from Abcam. Primary antibody for ELF3 (1:200) was provided by Thermo Scientific. Primary antibody for SNAI2 (1:200) were obtained from Cell Signaling Technology. H-Score system (range: 100–400) was used to assess the protein expression [20].

### Statistical analysis

GraphPad Prism 7.0 was used for statistical analysis. Data were expressed as mean ± SD. Student's *t* test or one way ANOVA was used to compare data among groups. Correlation analysis was performed using Pearson statistics. Survival was analyzed by Kaplan–Meier method. *P* value less that 0.05 was considered as significant.

## Results

### FZD5 maintains epithelial-like phenotype

**Fig. 1:**
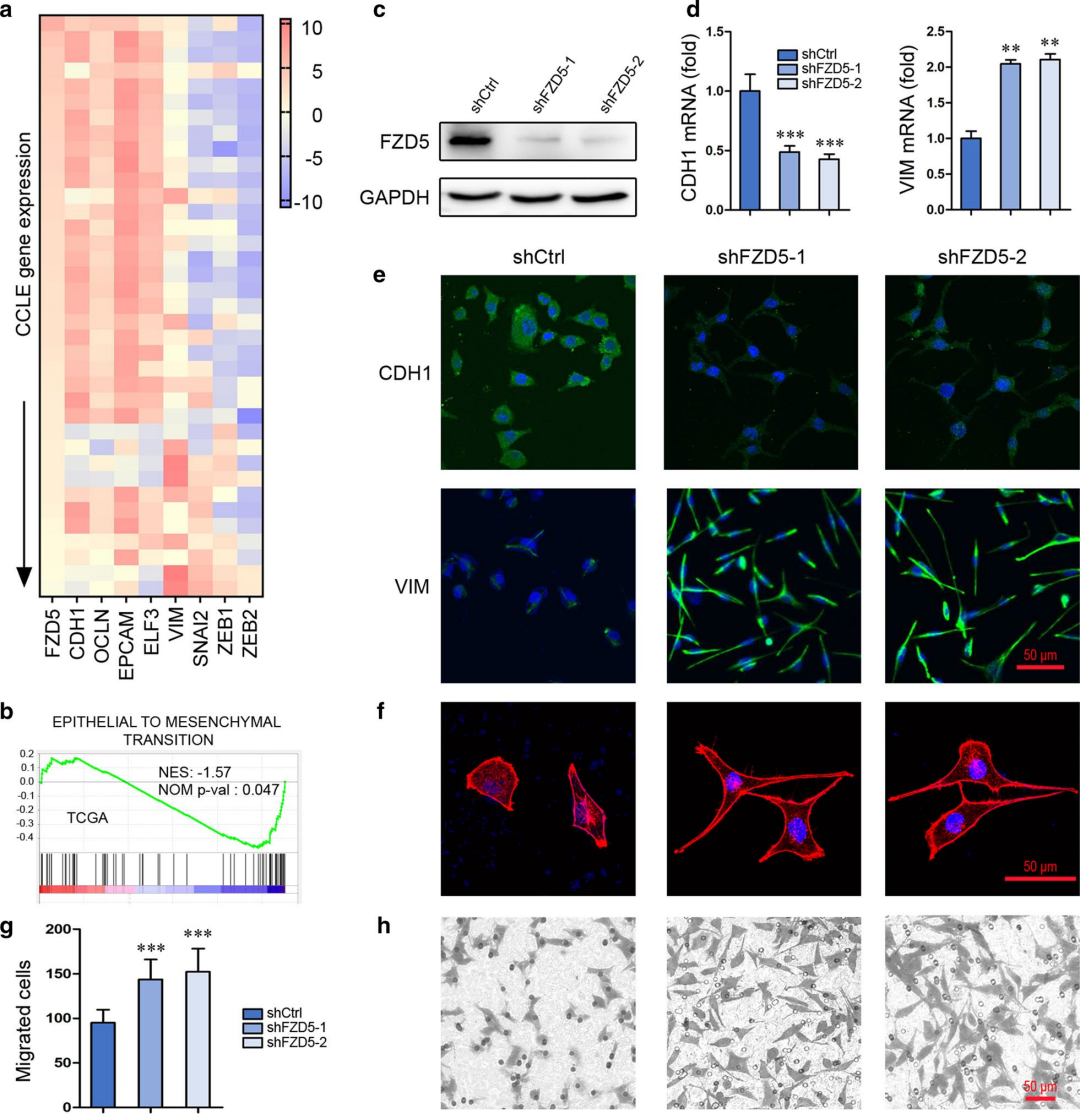
FZD5 maintains gastric cancer cell epithelial-like phenotype. **a** A heat map showing the correlation of FZD5 with EMT-related factors in gastric cancer cell lines was generated from CCLE database. **b** A pathway related to FZD5 in gastric cancer was shown by GO pathway enrichment analysis using TCGA database. **c** FZD5 expression in MKN45 cells was detected by Western blot. **d** and **e** CDH1 and Vim expression in MKN45 cells was detected by Real-time PCR and Immunofluorescence. **P < 0.01, ***P < 0.001, *vs* shCtrl. Scalebar: 50 μm. **f** MKN45 cells were stained with Phalloidin. Scalebar: 50 μm. **g** and **h** Migration of MKN45 cells was analyzed by Transwell. ***P < 0.001, *vs* shCtrl. Scalebar: 50 μm. All experiments were performed in triplicate

**Fig. 2:**
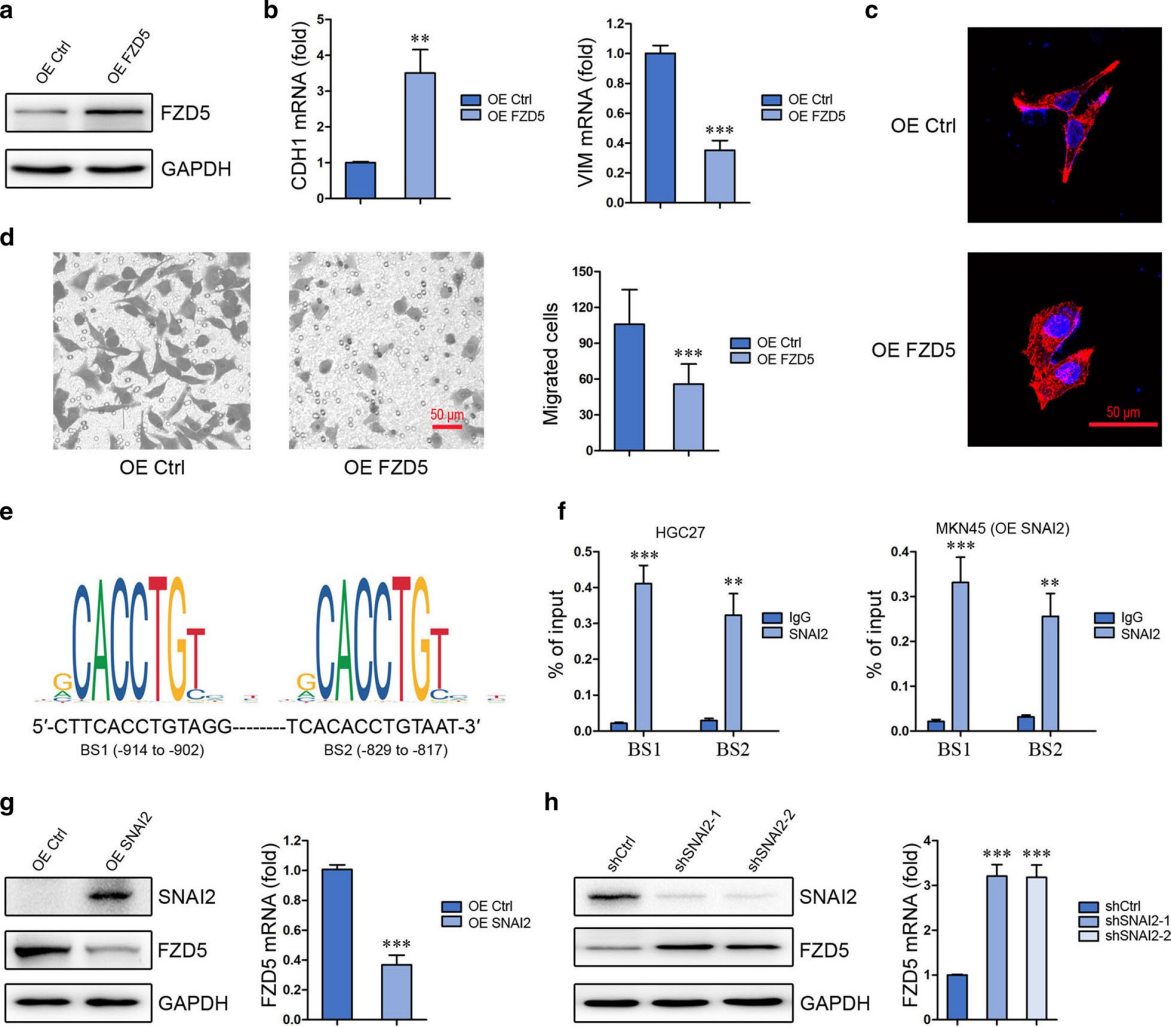
FZD5 maintains gastric cancer cell epithelial-like phenotype and transcriptionally modulated by SNAI2. **a** FZD5 expression in HGC27 cells was detected by Western blot. **b** CDH1 and Vim expression in HGC27 cells was detected by Real-time PCR. **P < 0.01, ***P < 0.001, *vs* OE Ctrl. **c** HGC27 cells were stained with Phalloidin. Scalebar: 50 μm. **d** Migration of HGC27 cells was analyzed by Transwell. ***P < 0.001, *vs* OE Ctrl. Scalebar: 50 μm. All experiments were performed in triplicate. **e** Binding sites of SNAI2 on FZD5 promoter were shown (http://jaspar.genereg.net/). **f** The binding of SNAI2 to FZD5 promoter in HGC27 and SNAI2-overexpressed MKN45 cells was analyzed by ChIP and real-time PCR. **P < 0.01, ***P < 0.001, *vs* IgG. **g** and **h** SNAI2 expression in MKN45 and HGC27 cells was detected by Western blot, and FZD5 expression in MKN45 and HGC27 cells was detected by Western blot and Real-time PCR. ***P < 0.001, *vs* OE Ctrl or shCtrl. All experiments were performed in triplicate

Interrogation of CCLE database revealed that FZD5 is positively correlated with epithelial-related factors CDH1 (E-cadherin), OCLN (Occludin), EPCAM and ELF3, while negatively correlated with mesenchymal-related factors VIM (Vimentin), SNAI2 (Slug), ZEB1 and ZEB2 in 37 gastric cancer cell lines (Fig. 1a). Pathway enrichment analysis of FZD5 using TCGA database showed that FZD5 is negatively associated with EMT in 375 gastric cancer tissues and 32 normal gastric tissues. (Fig. 1b). FZD5 knockdown in MKN45 cells downregulated CDH1 but upregulated VIM (Fig. 1c–e). Functionally, FZD5 knockdown in MKN45 cells induced a morphological alteration from epithelial-like to mesenchymal-like and an increase in cell migration (Fig. 1f–h). In contrast, FZD5 overexpression in HGC27 cells increased CDH1 but reduced VIM (Fig. 2a, b). Furthermore, FZD5 overexpression caused a morphological alteration from mesenchymal-like to epithelial-like and a decrease in cell migration (Fig. 2c, d). These results indicate that FZD5 maintains epithelial-like phenotype.

### SNAI2 transcriptionally inhibits FZD5

As FZD5 was shown to be inversely correlated with EMT transcription factor SNAI2 in mRNA level (Fig. 1a), whether SNAI2 modulates FZD5 transcription was investigated. Two binding sites (BS1 and BS2) of SNAI2 in FZD5 promoter were identified (Fig. 2e). ChIP in combination with Real-time PCR verified the binding of SNAI2 to these two sites in HGC27 cells and SNAI2-overexpressed MKN45 cells (Fig. 2f). SNAI2 overexpression in MKN45 cells reduced FZD5 expression in both mRNA and protein levels (Fig. 2g). Furthermore, SNAI2 knockdown in HGC27 cells induced FZD5 expression (Fig. 2h). These findings confirmed that SNAI2 negatively modulates FZD5.

### TEAD1 coordinates with SNAI2 in FZD5 transcription inhibition

**Fig. 3:**
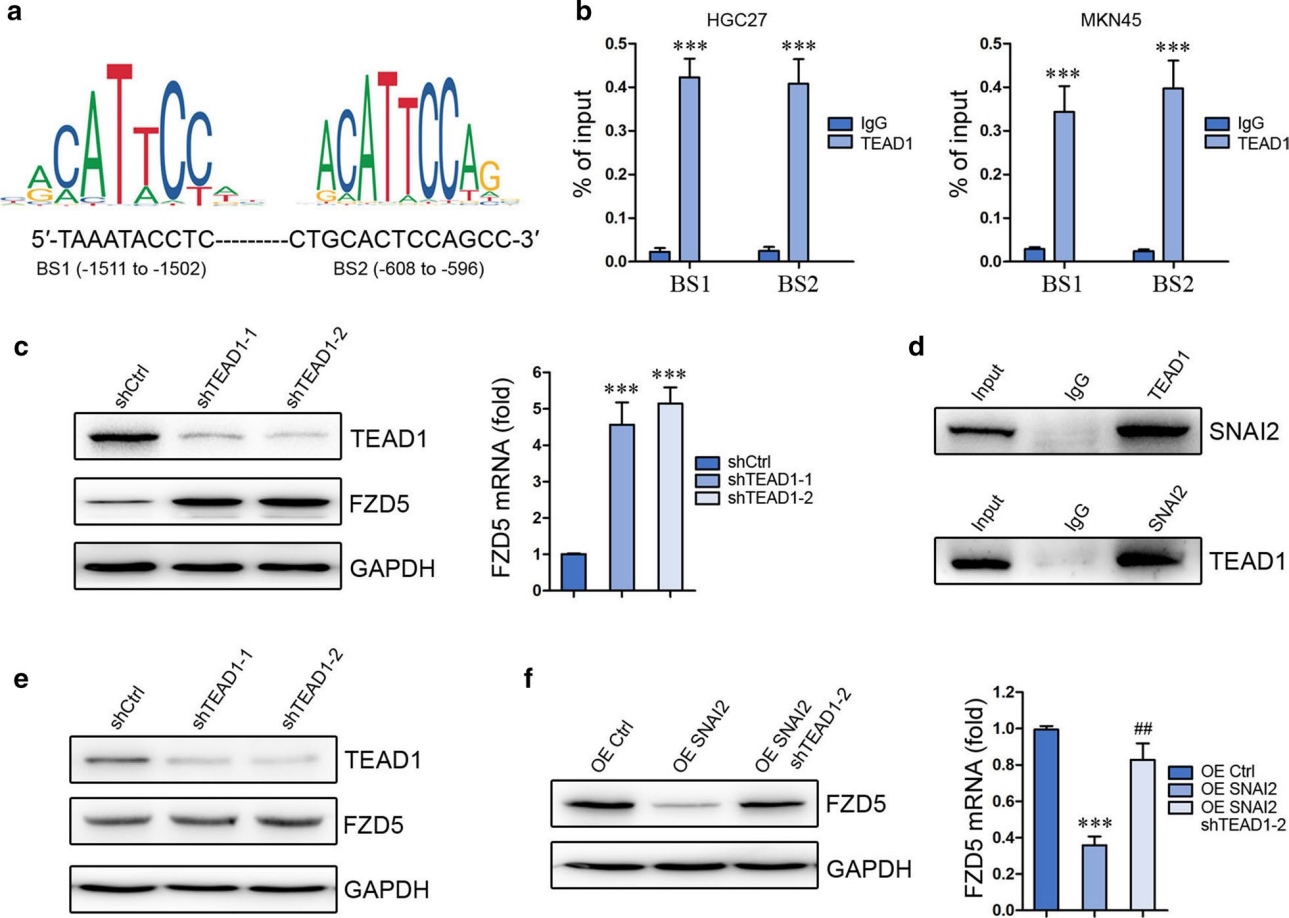
TEAD1 coordinates with SNAI2 to inhibit FZD5 transcription. **a** Binding sites of TEAD1 on FZD5 promoter were shown. **b** The binding of TEAD1 to FZD5 promoter in HGC27 and MKN45 cells was analyzed by ChIP and real-time PCR. ***P < 0.001, *vs* IgG. **c** TEAD1 expression in HGC27 cells was detected by Western blot, and FZD5 expression in HGC27 cells was detected by Western blot and Real-time PCR. ***P < 0.001, *vs* shCtrl. **d** Interaction of SNAI2 with TEAD1 in HGC27 cells was analyzed by CoIP. **e** TEAD1 and FZD5 expression in MKN45 cells was detected by Western blot. **f** FZD5 expression in MKN45 cells was detected by Western blot and Real-time PCR. ***P < 0.001, *vs* OE Ctrl; ##*P* < 0.01, *vs* OE SNAI2. All experiments were performed in triplicate

YAP/TEAD was shown to interact with SNAI2 in modulating gene transcription [21, 22]. Therefore, the role of TEAD1 in FZD5 transcription was subsequently evaluated. Two binding sites of TEAD1 in FZD5 promoter were identified, and the BS2 of TEAD1 is near the binding sites of SNAI2 (Fig. 3a). ChIP in combination with Real-time PCR verified the binding of TEAD1 to these two sites in HGC27 cells and MKN45 cells (Fig. 3b). TEAD1 knockdown in HGC27 cells upregulated FZD5 expression in both mRNA and protein levels (Fig. 3c). CoIP confirmed the interaction between SNAI2 and TEAD1 in HGC27 cells (Fig. 3d). TEAD1 knockdown in MKN45 cells had no effect on FZD5 expression (Fig. 3e), probably due to the absence of SNAI2 (Fig. 2g). However, TEAD1 knockdown in MKN45 cells blocked SNAI2 overexpression-induced FZD5 inhibition (Fig. 3f), demonstrating that TEAD1 coordinates with SNAI2 to modulate FZD5 transcription.

### ELF3 is a downstream effecter of FZD5

**Fig. 4:**
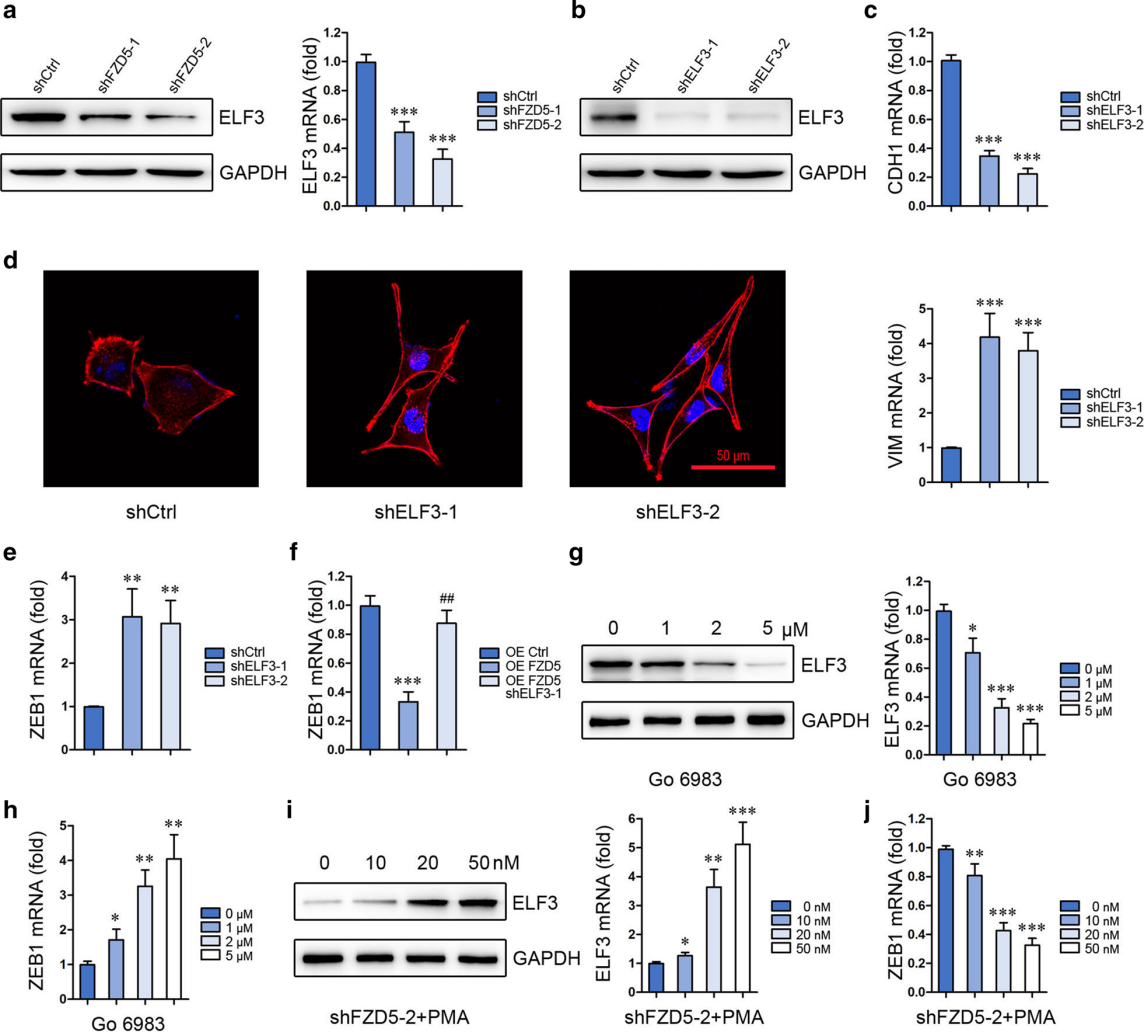
PKC-ELF3 is a downstream signaling of FZD5. **a** ELF3 expression in MKN45 cells was detected by Western blot and Real-time PCR. ***P < 0.001, *vs* shCtrl. **b** ELF3 expression in MKN45 cells was detected by Western blot. **c** CDH1 and Vim expression in MKN45 cells was detected by Real-time PCR. ***P < 0.001, *vs* shCtrl. **d** MKN45 cells were stained with Phalloidin. Scalebar: 50 μm. **e** ZEB1 mRNA level in MKN45 cells was detected by Real-time PCR. **P < 0.01, *vs* shCtrl. **f** ZEB1 mRNA level in HGC27 cells was detected by Real-time PCR. ***P < 0.001, *vs* OE Ctrl; ##*P* < 0.01, *vs* OE FZD5. **g** ELF3 expression in MKN45 cells was detected by Western blot and Real-time PCR. *P < 0.05, ***P < 0.001, *vs* 0 μM. **h** ZEB1 mRNA level in MKN45 cells was detected by Real-time PCR. *P < 0.05, **P < 0.01, *vs* 0 μM. **i** ELF3 expression in MKN45 cells was detected by Western blot and Real-time PCR. *P < 0.05, **P < 0.01, ***P < 0.001, *vs* 0 nM. **j** ZEB1 mRNA level in MKN45 cells was detected by Real-time PCR. **P < 0.01, ***P < 0.001, *vs* 0 nM. All experiments were performed in triplicate

As shown in Fig. 1a, FZD5 is positively correlated with ELF3, an epithelial-specific factor. FZD5 knockdown significantly inhibited ELF3 expression, indicating that ELF3 is a downstream molecule of FZD5 (Fig. 4a). Similar to FZD5 knockdown, ELF3 knockdown in MKN45 cells altered CDH1 and VIM expression and cell morphology (Fig. 4b–d). Mechanistically, ELF3 knockdown increased ZEB1 mRNA level in MKN45 cells (Fig. 4e). Furthermore, ELF3 knockdown in HGC27 cells blocked FZD5 overexpression-induced ZEB1 transcription downregulation (Fig. 4f). The linker between FZD5 and ELF3 was then explored. PKC was shown to be a downstream molecule of FZD5 and a modulator of ELF3 [23–26]. Treatment with PKC inhibitor Go6983 repressed ELF3 expression (Fig. 4g), and stimulated ZEB1 transcription in MKN45 cells (Fig. 4h). Treatment with PKC activator PMA upregulated ELF3 expression and inhibits ZEB1 transcription in shFZD5-transfected MKN45 cells (Fig. 4i, j). These findings establish a FZD5-PKC-ELF3-ZEB1 pathway in gastric cancer.

### LRP5 is required for FZD5 signaling

**Fig. 5:**
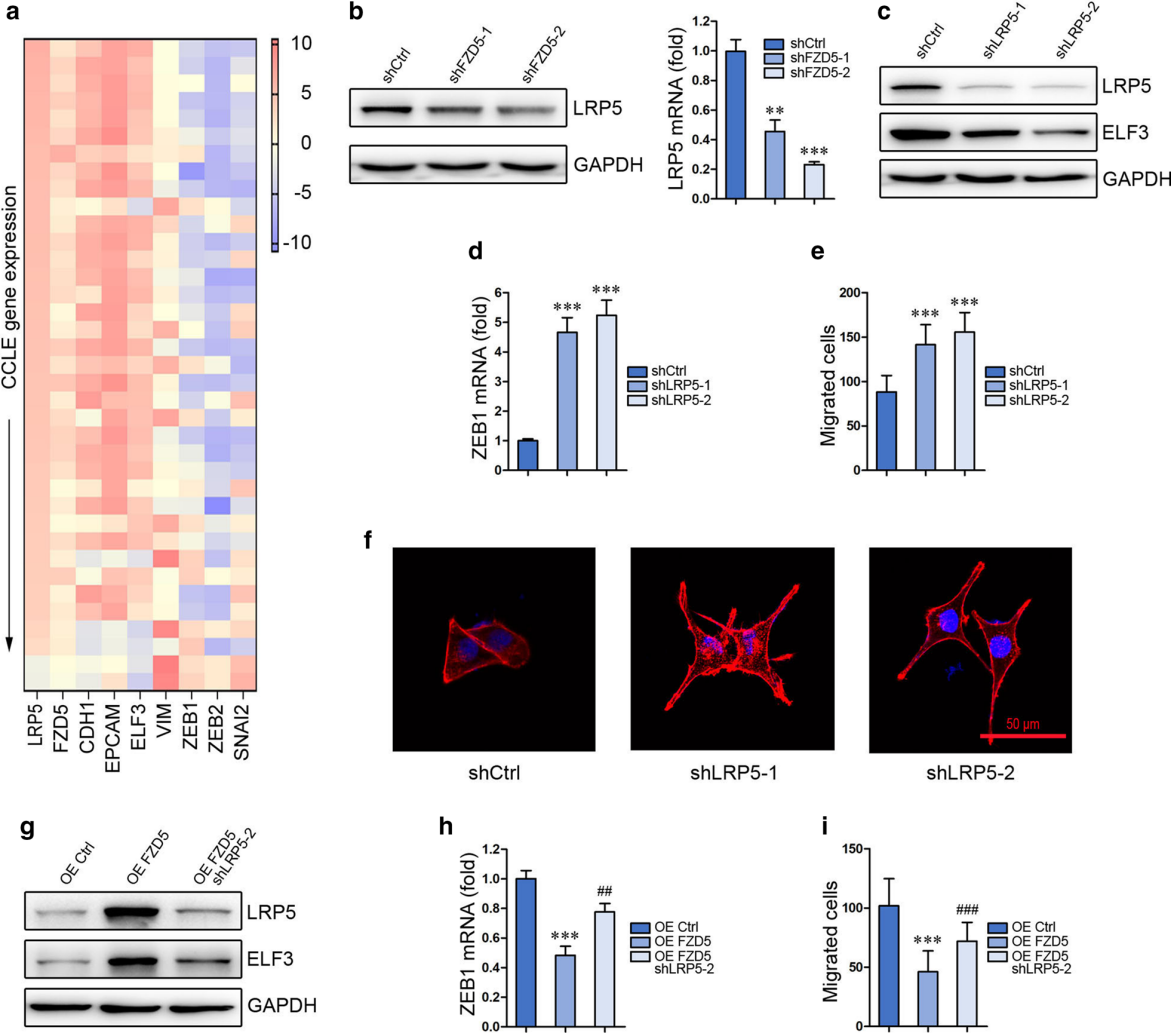
FZD5 signaling requires its co-receptor LRP5. **a** A heat map showing the correlation of LRP5 with FZD5 and EMT-related factors in gastric cancer cell lines was generated from CCLE database. **b** LRP5 expression in MKN45 cells was detected by Western blot and Real-time PCR. **P < 0.01, ***P < 0.001, *vs* shCtrl. **c** LRP5 and ELF3 expression was detected by Western blot in MKN45 cells. **d** ZEB1 mRNA level in MKN45 cells was detected by Real-time PCR. ***P < 0.001, *vs* shCtrl. **e** Migration of MKN45 cells was analyzed by Transwell. ***P < 0.001, *vs* shCtrl. Scalebar: 50 μm. **f** MKN45 cells were stained with Phalloidin. Scalebar, 50 μm. **g** LRP5 and ELF3 expression in HGC27 cells was detected by Western blot. **h** ZEB1 mRNA level in HGC27 cells was detected by Real-time PCR. ***P < 0.001, *vs* OE Ctrl; ##*P* < 0.01, *vs* OE FZD5. **i** Migration of HGC27 cells was analyzed by Transwell. ***P < 0.001, *vs* OE Ctrl; ###*P* < 0.001, *vs* OE FZD5. Scalebar: 50 μm. All experiments were performed in triplicate

LRP5 is a co-receptor of FZDs. Interestingly, interrogation of CCLE database revealed that LRP5 is positively correlated with FZD5, CDH1, EPCAM and ELF3, while negatively correlated with VIM, SNAI2, ZEB1 and ZEB2 (Fig. 5a). FZD5 knockdown in MKN45 cells reduced LRP5 expression, indicating LRP5 is modulated by FZD5 (Fig. 5b). The role of LRP5 in FZD5 signaling and EMT was subsequently investigated. LRP5 knockdown in MKN45 cells repressed ELF3 expression but promoted ZEB1 transcription and cell migration (Fig. 5c–e). LRP5 knockdown in MKN45 cells also induced a morphological alteration from epithelial-like to mesenchymal-like (Fig. 5f). Furthermore, LRP5 knockdown in HGC27 cells blocked the effects of FZD5 overexpression on ELF3 expression, ZEB1 transcription and cell migration (Fig. 5g–i). These findings demonstrate that FZD5 signaling in modulating EMT requires its co-receptor LRP5.

### WNT7B is a putative ligand for FZD5

**Fig. 6:**
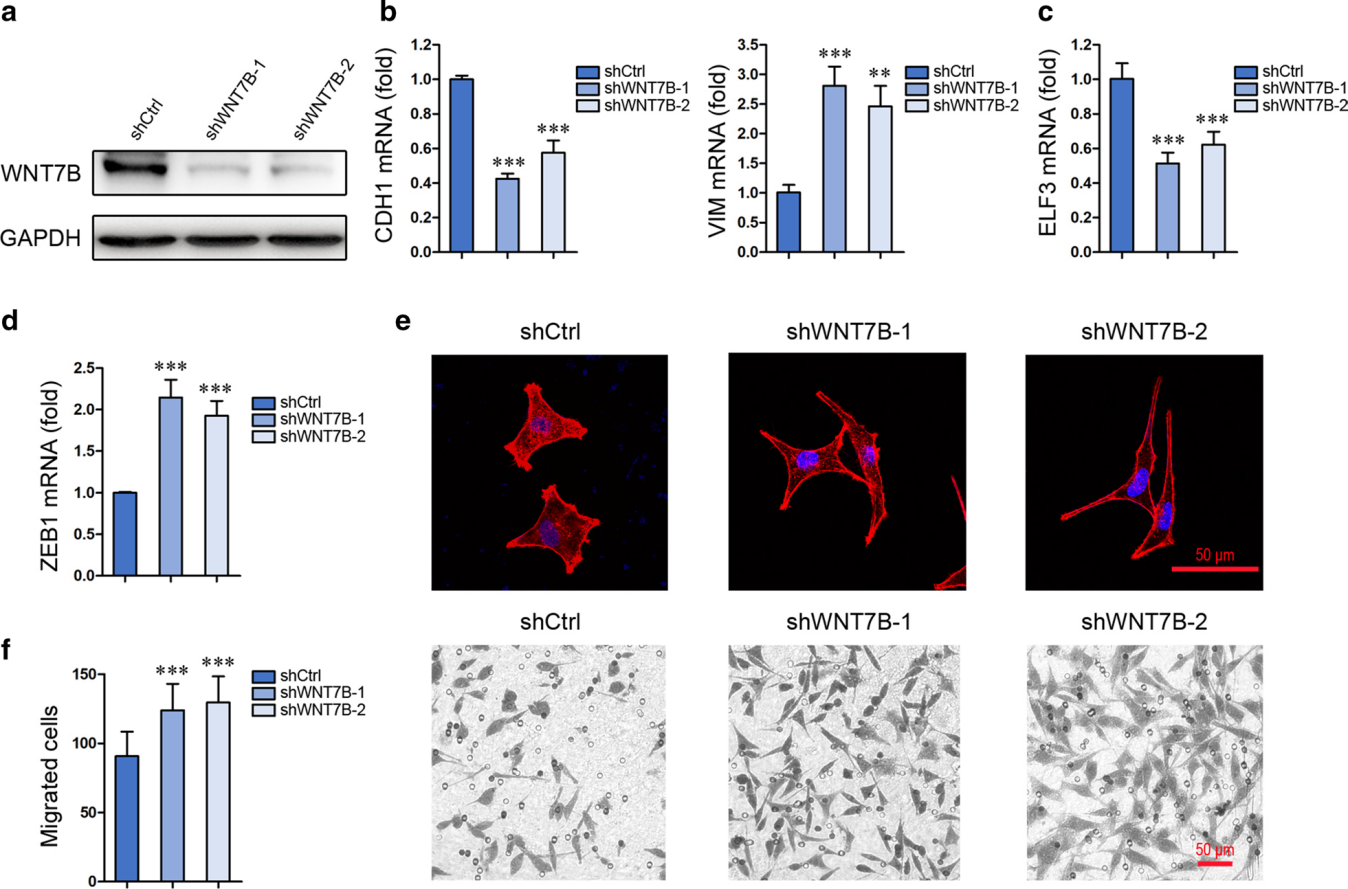
WNT7B is a putative ligand for FZD5. **a** WNT7B expression in MKN45 cells was detected by Western blot. **b** CDH1 and Vim expression in MKN45 cells was detected by Real-time PCR. **P < 0.01, ***P < 0.001, *vs* shCtrl. **c** and **d** ELF3 and ZEB1 expression in MKN45 cells was detected by Real-time PCR. ***P < 0.001, *vs* shCtrl. **e** MKN45 cells were stained with Phalloidin. Scalebar: 50 μm. **f** Migration of MKN45 cells was analyzed by Transwell. ***P < 0.001, *vs* shCtrl. Scalebar, 50 μm. All experiments were performed in triplicate

Just like FZD5 knockdown, WNT7B knockdown in MKN45 cells inhibited CDH1 expression but induced VIM expression (Fig. 6a, b). Moreover, WNT7B knockdown in MKN45 cells reduced ELF3 expression but increased ZEB1 transcription (Fig. 6c, d). Similarly, WNT7B knockdown in MKN45 cells induced a morphological alteration from epithelial-like to mesenchymal-like and an increase in cell migration (Fig. 6e, f).

### FZD5 signaling is related to patient survival

**Fig. 7:**
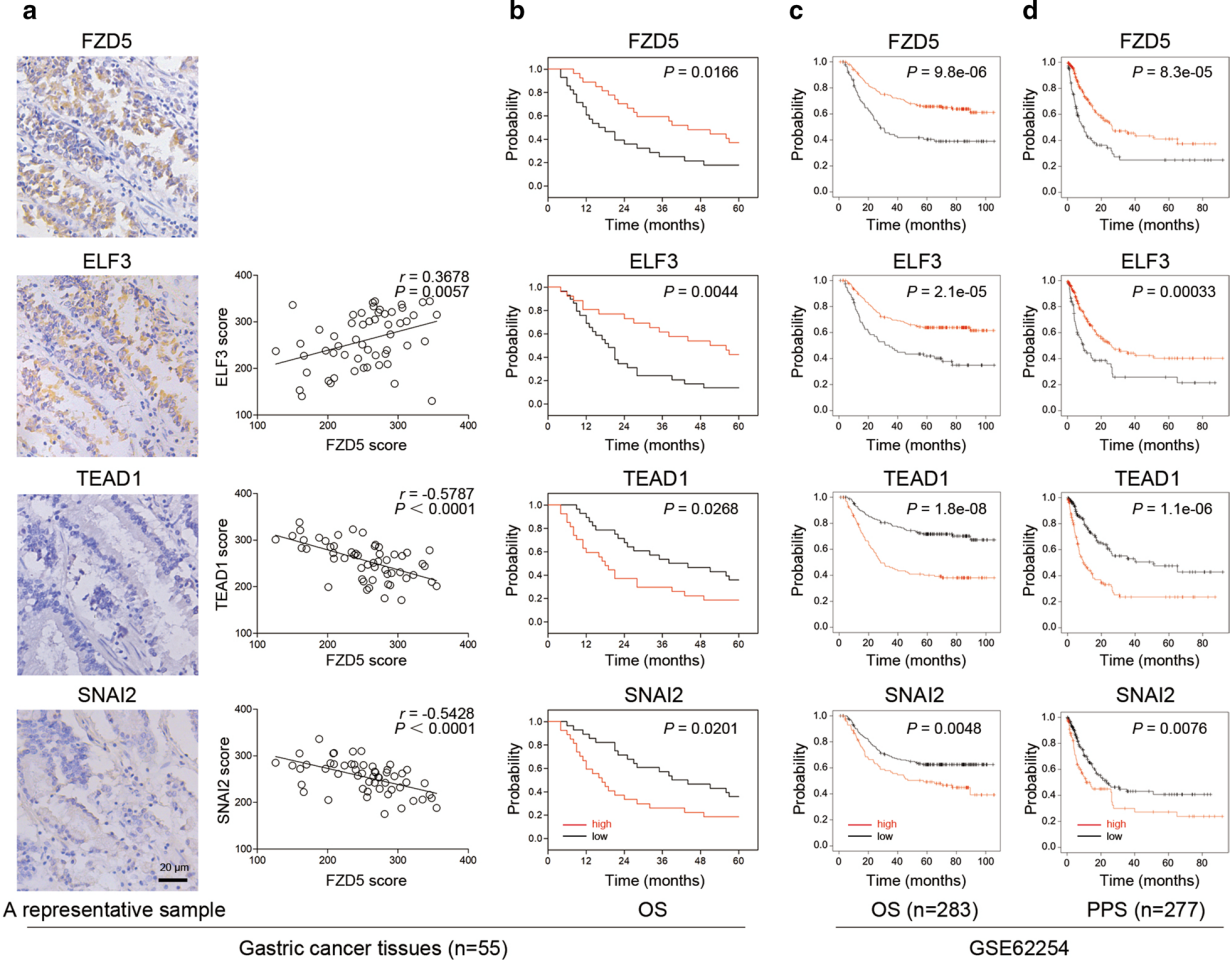
FZD5 signaling is related to patient survival. **a** FZD5, ELF3, TEAD1 and SNAI2 expression in 55 GC tissues was detected by Immunohistochemistry. Protein expression was scored by H-score system. Staining of a representative sample was shown. Scalebar: 20 μm. Correlation of FZD5 with ELF3, SNAI2 and TEAD1 was analyzed by Pearson statistics. **b** The association of FZD5, ELF3, SNAI2 and TEAD1 with OS in 55 GC tissues was analyzed by Kaplan–Meier method. **c** The association of FZD5, ELF3, SNAI2 and TEAD1 with OS was analyzed using GSE62254 database in Kaplan Meier plotter website. **d** The association of FZD5, ELF3, SNAI2 and TEAD1 with PPS was analyzed using GSE62254 database in Kaplan Meier plotter website

FZD5, ELF3, TEAD1 and SNAI2 expression in 55 gastric cancer tissues was detected by immunohistochemistry. Expression scoring showed that FZD5 is positively correlated with ELF3 while negatively correlated with TEAD1 and SNAI2 (Fig. 7a). EMT potentially promotes tumor metastasis, leading to unfavorable clinical outcomes. Accordingly, the association of these factors with survival was analyzed. High FZD5 and ELF3 protein expression is correlated with longer overall survival (OS); in contrast, high TEAD1 and SNAI2 protein expression is correlated with shorter survival (Fig. 7b). GSE62254 database was interrogated to assess the association of these factors with survival in mRNA levels [18, 19]. Consistently, high FZD5 and ELF3 mRNA expression is related to longer survival, while high TEAD1 and SNAI2 mRNA expression is related to shorter survival (Fig. 7c, d). These findings suggest that FZD5-ELF3 signaling contributes to good clinical outcome in gastric cancer.

## Discussion

Among 19 WNT ligands in human, WNT7A/B and WNT5A have been reported to bind to FZD5. WNT7A-FZD5 signaling promotes endometrial and ovarian cancer cell proliferation and growth through β-catenin pathway [27, 28]. Similarly, WNT7B-FZD5 signaling promotes RNF43-mutant pancreatic cancer cell proliferation and growth [29]. Upon binding WNT5A, FZD5 mediates β-catenin-independent pathways. WNT5A-FZD5 signaling increases cell motility in melanoma and classical Hodgkin lymphoma [3, 30]. Together, these findings demonstrate FZD5 as a promoter in some human tumors.

However, our study revealed that FZD5 is a putative suppressor in gastric cancer. Interrogation of databases showed an association of FZD5 with epithelial-like phenotype. Loss and gain of function studies further confirmed that FZD5 prevents cancer cell EMT. Prognosis analysis indicated that high FZD5 expression is correlated with longer survival period. Furthermore, our study showed that WNT7B is a putative ligand for FZD5 in gastric cancer. WNT7A/B seems to specifically bind to FZD5 among 10 FZDs [31]. Actually, WNT7A functions as an EMT inhibitor in lung cancer [32]. Moreover, interrogation of CCLE database showed that FZD5 is also associated with epithelial-like phenotype in lung cancer (data not shown).

As shown in our study, FZD5 is transcriptional inhibited by TEAD1-SNAI2 complex. Activation of tumor-suppressing Hippo pathway results in phosphorylation and degradation of Yap and Taz. When Yap and Taz are not phosphorylated, they translocate into the nucleus to bind transcription factor TEAD. Yap/Taz-TEAD signaling induces gastric cancer cell EMT, stemness, drug resistance and metastasis [33–37]. Intriguingly, Yap/Taz is modulated by WNT5A [7, 8, 38, 39], suggesting that FZD5 signaling may be suppressed by WNT5A in gastric cancer. In addition, TEAD1 and SNAI2 coordinate to modulate gene transcription in both physiological and pathological processes [21, 22, 40].

ELF3 is a universal epithelial-limited transcription factor. Similar to FZD5, ELF3 has dual roles in human tumor. ELF3 induces breast epithelial cell malignant transformation [41, 42], and promotes prostate cancer progression by interacting with NFκB [43]. In gastric cancer, ELF3 maintains epithelial-like phenotype, prevents EMT and is associated with longer survival, acting as a putative tumor suppressor. ELF3 also inhibits EMT in ovarian and breast cancer cells [44, 45]. Mechanistically, ELF3 represses activity and expression of EMT transcription factor ZEB1 [46, 47].

Activation of β-catenin pathway requires not only FZDs, but also their co-receptors LRP5/6 which is not involved in non-canonical β-catenin-independent pathway. A recent study reported that FZDs-LRP5/6 interaction can initiate β-catenin pathway in the absence of WNTs [48]. Surprisingly, another study revealed that FZDs-LRP5/6 interaction can block activation of FZD-mediated non-canonical pathway, further preventing tumor metastasis [49]. These findings, in combination with our observations, suggest that FZDs-LRP5/6 interaction may act as a tumor promoter by activating β-catenin pathway or a tumor suppressor by antagonizing β-catenin-independent pathway, depending on tumor or cell context.

## Conclusions

In summary, our study for the first time uncovers that FZD5-ELF3 signaling prevents EMT and is associated with favorable prognosis in gastric cancer. Therefore, FZD5 and ELF3 function as putative tumor suppressors in this type of cancer. Antibodies against FZD5 including ipafricept and vantictumab have been shown effective on certain cancer types [50]. However, our study warns out that targeting FZD5 should be cautious and tumor-specific, since treatment with these antibodies may potentially promote metastasis in some other types of cancers including gastric cancer.

## Data Availability

All data generated or analysed during this study are included in this published article.

## Abbreviations

FZD: Frizzled
EMT: Epithelial-mesenchymal transition
PKC: Protein kinase C
GPCRs: G protein-coupled receptors
CCLE: Cancer Cell Line Encyclopedia
TCGA: The Cancer Genome Atlas
ChIP: Chromatin Immunoprecipitation
CoIP: Co-immunoprecipitation
LRP5: LDL receptor-related proteins 5
